# Supplementary material for: Neurobehavioral Signatures in Overgrowth Intellectual Disability Syndromes: Dissecting Genotype-Phenotype Relationships in the PI3K-AKT-MTOR Pathway
Source: medRxiv. 2025 Aug 24:2025.08.19.25333388. Preprint. [Version 1] doi: 10.1101/2025.08.19.25333388 (PMC12393652; doi:10.1101/2025.08.19.25333388)
Supplement: Supplement 1 [file media-1.pdf]

Supplement

Table S1. Neurobehavioral Battery Adapted from the PHTS Developmental Synaptopathies Consortium

Measure	Domain
Social Responsiveness Scale 2 (SRS-2)	ASD-like Behaviors
Repetitive Behavioral Scale Revised (RBS-R)	Repetitive Behaviors
Child Behavior Checklist Adult/Observer Report Form (CBCL/ARF)	Behavioral Functioning
Aberrant Behavior Checklist – Community (ABC-C)[a]	Aberrant Behaviors
Vineland Adaptive Behavior Scale (VABS-II)	Adaptive Functioning
Developmental Coordination Disorder Questionnaire (DCDQ)	Motor Functioning
Behavior Rating Inventory of Executive Function (BRIEF)	Executive Functioning
Short Sensory Profile (SSP)	Sensory Functioning

[a] Not included in original DSC battery

Table S2. Descriptive Characteristics of Study Cohorts

Cohort	N	Median Age (Months)	Age IQR	Female Proportion
Healthy Control	30	156	105-207.8	0.53
SKS	17	57	45-97	0.24
Macrocephaly ASD	32	126	89.3-204.8	0.16
PTEN no-ASD	28	104	67.75-132	0.36
PTEN-ASD	43	107	66-140.5	0.21

IQR = Interquartile range; N = number of participants

Table S3. Neurobehavioral Profile of SKS and PTEN Patients

Measure	SKS median	SKS IQR	PTEN ASD median	PTEN ASD IQR	Macrocephaly ASD median	Macrocephaly ASD IQR	PTEN No-ASD Median	PTEN no-ASD IQR	Healthy Control median	Healthy Control IQR
ABC Hyperactivity	12	8.8-15.2	9	6.0-16.8	13	8.5-19.5	4	1.0-9.2	NA	NA
ABC Irritability	11	7.0-15.2	5	1.2-11.0	10	4.0-13.0	1	0.0-5.2	NA	NA
ABC Lethargy	9.5	4.0-12.0	7.5	2.0-18.0	10	7.5-14.5	1	0.0-5.5	NA	NA
ABC Speech	1	0.0-3.2	3	2.0-5.0	3	2.0-4.0	1	0.0-2.2	NA	NA
ABC Stereotypies	7	3.0-9.5	4.5	1.0-8.0	5	2.0-8.5	0	0.0-1.2	NA	NA
ABC Total Score	42	31.8-53.8	32	19.2-57.2	39	33.5-50.5	13	5.0-22.8	NA	NA
BRIEF Emotional Control	56	55.0-64.0	57	50.5-64.2	64	55.5-73.2	50.5	39.5-57.2	44.5	37.0-50.2
BRIEF Inhibit	58	52.0-63.0	60	54.5-69.2	69	62.2-72.8	46.5	44.8-57.8	45	41.0-49.0
BRIEF Plan/Organize	57	46.0-68.0	65.5	51.8-76.0	71.5	61.8-75.0	51	42.5-70.0	42	38.8-58.8
BRIEF Shift	60	45.0-69.0	62.5	49.8-70.2	68	59.8-74.0	53	45.2-56.0	40.5	38.8-49.2
BRIEF Total	63	56.0-73.0	66	56.5-76.0	73	65.2-78.0	53	42.0-73.5	42.5	37.8-53.2
BRIEF Working Memory	68	66.0-74.0	73	59.5-78.5	71.5	62.2-79.8	56.5	47.2-75.2	45	40.0-49.2
CBCL Externalizing	57	47.5-64.0	53.5	43.0-60.0	58	48.0-63.8	41	36.5-43.5	48.5	34.0-53.2
CBCL Internalizing	59	57.2-64.2	63	55.0-67.2	61.5	52.0-67.5	48	37.5-63.5	48	34.0-55.2
CBCL Total	60.5	58.0-67.2	63.5	52.5-71.0	66.5	59.2-70.0	46	44.5-60.5	45.5	34.0-53.2
DCDQ Control	6	6.0-9.0	9	8.0-13.0	14.5	10.2-15.8	14	10.0-23.0	30	25.5-30.0
DCDQ Coordination	7	5.0-8.0	8	6.0-11.0	10	7.2-12.0	14	11.0-18.5	22	18.5-25.0
DCDQ Fine Motor	4	4.0-4.0	5	4.0-8.0	7.5	4.2-12.8	10	7.0-14.0	20	18.5-20.0
DCDQ Total Score	17.5	15.0-20.8	23	21.0-34.0	33.5	25.5-41.5	38	27.5-54.0	70	64.0-75.0
RBS-R Total Score	19	14.5-38.0	20.5	11.0-46.5	26.5	15.5-33.2	3.5	1.0-15.2	NA	NA
SRS-2 Total Score	75	70.0-85.0	75.5	68.5-83.0	77	71.0-81.8	47	44.0-65.0	43	40.5-47.0
SSP Auditory Filtering	18	15.8-20.0	18	14.2-21.5	18	16.0-19.8	23	20.0-27.0	28	23.8-30.0
SSP Low Energy	15.5	14.0-18.2	15	8.0-21.8	23	16.5-28.0	20	14.8-27.5	30	29.0-30.0
SSP Movement	14.5	11.0-15.0	11.5	9.0-15.0	14	12.0-15.0	13	10.0-15.0	15	14.0-15.0

SSP Tactile	27.5	23.0-29.3	28	24.0-31.8	28.5	27.0-32.0	32	30.8-35.0	35	33.0-35.0
SSP Taste/Smell	18.5	11.8-20.0	16	7.2-20.0	17.5	12.0-20.0	19	15.8-20.0	20	16.8-20.0
SSP Total	124	111.4-136.0	120	102.8-140.8	140	126.0-149.0	154.5	134.8-172.8	185	163.0-188.5
SSP Underresponsive/Seeks	20	16.0-22.0	20.5	15.5-27.0	22.5	19.2-27.5	31.5	22.8-34.2	35	29.8-35.0
SSP Visual/Auditory	16	13.0-20.2	16	13.0-19.0	18	14.2-20.8	21	17.8-24.0	25	18.0-25.0
VABS-II Adaptive Behavior	53.5	46.2-59.8	62	55.0-75.2	61	55.0-70.0	95.5	80.2-111.8	94	88.5-109.8
VABS-II Communication	59	42.0-65.0	64	56.0-78.0	66	57.5-74.2	92.5	83.5-113.5	100	91.2-111.0
VABS-II Daily Living	52	46.0-56.0	66	58.0-77.0	66	56.5-73.0	101	81.5-109.8	96	87.5-106.5
VABS-II Motor	51	37.0-61.0	68.5	61.0-75.8	67	61.0-91.0	78	70.0-91.0	111	102.0-115.5
VABS-II Socialization	61	57.0-68.0	68.5	56.5-76.8	59	48.0-69.0	104.5	88.0-119.0	94.5	85.0-103.0

Values shown as median (interquartile range). SKS: MTOR-associated Smithsonian-Kingsmore Syndrome; PTEN-ASD: PTEN-associated autism spectrum disorder. Measures: SRS-2 (Social Responsiveness Scale-2; higher scores indicate greater social impairment), RBS-R (Repetitive Behavior Scale-Revised; higher scores indicate more repetitive behaviors), VABS-II (Vineland Adaptive Behavior Scales-II; lower scores indicate greater impairment), CBCL (Child Behavior Checklist; higher scores indicate more behavioral problems), DCDQ (Developmental Coordination Disorder Questionnaire; lower scores indicate greater motor impairment), ABC (Aberrant Behavior Checklist; higher scores indicate more problematic behaviors), BRIEF (Behavior Rating Inventory of Executive Function; higher scores indicate greater executive function difficulties), SSP (Short Sensory Profile; lower scores indicate greater sensory differences).

Table S4. Neurobehavioral Feature Comparison Between SKS and Healthy Controls

Domain	Measure	N SKS	N Healthy Control	W	p-value	Effect Size (r)	p-value adj	Sig	Sig adj
Adaptive Function	VABS-II Adaptive Behavior	22	10	198	3.70E-04	0.63	4.60E-04	***	***
Adaptive Function	VABS-II Communication	22	13	270	1.50E-05	0.73	7.70E-05	***	***
Adaptive Function	VABS-II Daily Living	22	12	241	9.10E-05	0.67	2.30E-04	***	***
Adaptive Function	VABS-II Motor	15	9	120	0.002	0.63	0.002	**	**
Adaptive Function	VABS-II Socialization	22	11	220.5	1.50E-04	0.66	2.60E-04	***	***
Autism Symptoms	SRS-2 Total Score	20	16	1.5	4.80E-07	0.84	4.80E-07	***	***
Behavior	CBCL Externalizing	24	16	96	0.008	0.42	0.008	**	**
Behavior	CBCL Internalizing	24	16	73	0.001	0.52	0.002	**	**
Behavior	CBCL Total	24	16	48	7.30E-05	0.63	2.20E-04	***	***
Executive Function	BRIEF Emotional Control	24	13	77.5	0.013	0.41	0.015	*	*
Executive Function	BRIEF Inhibit	24	13	59	0.002	0.51	0.003	**	**
Executive Function	BRIEF Plan/Organize	24	13	99	0.072	0.3	0.072		
Executive Function	BRIEF Shift	24	13	60	0.002	0.5	0.003	**	**
Executive Function	BRIEF Total	24	13	55	0.001	0.53	0.003	**	**
Executive Function	BRIEF Working Memory	24	13	27	4.10E-05	0.67	2.50E-04	***	***
Motor	DCDQ Control	23	16	366.5	7.10E-08	0.86	1.40E-07	***	***
Motor	DCDQ Coordination	23	16	368	1.10E-07	0.85	1.40E-07	***	***
Motor	DCDQ Fine Motor	23	16	368	3.20E-08	0.89	1.30E-07	***	***
Motor	DCDQ Total Score	23	16	368	1.40E-07	0.84	1.40E-07	***	***
Sensory	SSP Auditory Filtering	24	16	344.5	2.20E-05	0.67	3.50E-05	***	***
Sensory	SSP Low Energy	24	16	384	3.60E-08	0.87	2.90E-07	***	***
Sensory	SSP Movement	24	16	241	0.123	0.24	0.123		
Sensory	SSP Tactile	24	16	345.5	1.70E-05	0.68	3.30E-05	***	***
Sensory	SSP Taste/Smell	24	16	246	0.101	0.26	0.115		
Sensory	SSP Total	24	15	354	5.20E-07	0.8	2.10E-06	***	***
Sensory	SSP Underresponsive/Seeks	24	15	338	3.50E-06	0.74	9.40E-06	***	***

Sensory	SSP Visual/Auditory	24	16	322	2.50E-04	0.58	3.40E-04	***	***

Mann-Whitney U tests were performed to compare behavioral measures between individuals with MTOR-associated Smithsonian-Kingsmore Syndrome (SKS) and healthy controls, with false discovery rate adjustment (adj) correction applied within each domain. N SKS and N Healthy Controls indicate number of subjects with data available for each measure. W: Mann-Whitney U test statistic; r: effect size. Measures: SRS-2 (Social Responsiveness Scale-2), RBS-R (Repetitive Behavior Scale-Revised), VABS-II (Vineland Adaptive Behavior Scales-II), CBCL (Child Behavior Checklist), DCDQ (Developmental Coordination Disorder Questionnaire), ABC (Aberrant Behavior Checklist), BRIEF (Behavior Rating Inventory of Executive Function), SSP (Short Sensory Profile). *p<0.05, **p<0.01, ***p<0.001.

Table S5. Neurobehavioral Feature Comparison Between SKS and PTEN-ASD

Domain	Measure	N SKS	N PTEN-ASD	W	p-value	Effect size (r)	p-value adj	Sig	Sig adj
Aberrant Behavior	ABC Hyperactivity	16	38	356	0.328	0.13	0.418		
Aberrant Behavior	ABC Irritability	16	38	404	0.059	0.26	0.176		
Aberrant Behavior	ABC Lethargy	16	38	299	0.932	0.01	0.932		
Aberrant Behavior	ABC Speech	16	38	199	0.044	0.27	0.176	*	
Aberrant Behavior	ABC Stereotypies	16	38	377	0.168	0.19	0.336		
Aberrant Behavior	ABC Total Score	16	38	354	0.348	0.13	0.418		
Adaptive Function	VABS-II Adaptive Behavior	10	36	105	0.047	0.29	0.079	*	
Adaptive Function	VABS-II Communication	13	37	165	0.097	0.23	0.121		
Adaptive Function	VABS-II Daily Living	12	37	108	0.008	0.38	0.041	**	*
Adaptive Function	VABS-II Motor	9	18	38.5	0.03	0.42	0.076	*	
Adaptive Function	VABS-II Socialization	11	36	172.5	0.53	0.09	0.53		
Autism Symptoms	RBS-R Total Score	16	38	303	0.992	0	0.992		
Autism Symptoms	SRS-2 Total Score	16	38	349	0.399	0.11	0.797		
Behavior	CBCL Externalizing	16	16	155	0.317	0.18	0.952		
Behavior	CBCL Internalizing	16	16	120.5	0.792	0.05	0.97		
Behavior	CBCL Total	16	16	129.5	0.97	0.01	0.97		
Executive Function	BRIEF Emotional Control	13	32	208	1	0	1		
Executive Function	BRIEF Inhibit	13	32	165	0.286	0.16	0.859		
Executive Function	BRIEF Plan/Organize	13	32	153	0.172	0.2	0.859		
Executive Function	BRIEF Shift	13	32	185	0.573	0.08	0.87		
Executive Function	BRIEF Total	13	31	179.5	0.58	0.08	0.87		
Executive Function	BRIEF Working Memory	13	32	194.5	0.744	0.05	0.893		
Motor	DCDQ Control	16	37	160.5	0.008	0.36	0.011	**	*
Motor	DCDQ Coordination	16	37	192	0.042	0.28	0.042	*	*
Motor	DCDQ Fine Motor	16	37	106	7.70E-05	0.54	3.10E-04	***	***
Motor	DCDQ Total Score	16	37	125.5	9.50E-04	0.45	0.002	***	**

Sensory	SSP Auditory Filtering	16	38	301.5	0.97	0.01	0.97
Sensory	SSP Low Energy	16	38	319	0.783	0.04	0.97
Sensory	SSP Movement	16	38	396.5	0.074	0.24	0.592
Sensory	SSP Tactile	16	38	280	0.656	0.06	0.97
Sensory	SSP Taste/Smell	16	38	374.5	0.175	0.18	0.699
Sensory	SSP Total	15	38	291	0.913	0.01	0.97
Sensory	SSP Underresponsive/Seeks	15	38	237.5	0.353	0.13	0.941
Sensory	SSP Visual/Auditory	16	38	320.5	0.761	0.04	0.97

Mann-Whitney U tests were performed to compare behavioral measures between individuals with SKS and PTEN-associated autism spectrum disorder (PTEN-ASD), with FDR correction applied within each domain (adj). N SKS and N PTEN-ASD indicate number of subjects with data available for each measure. r: effect size; W: Mann-Whitney U test statistic;. Domain measures as defined in Table 1. *p<0.05, **p<0.01, ***p<0.001

Table S6. MANOVA results comparing SKS, PTEN-ASD, and Macrocephaly ASD groups

Domain	Total N	Pillai	F	df	p-value	Sig
Adaptive Function	40	0.47	2.1	10, 68	0.036	*
Autism Symptoms	79	0.06	1.19	4, 152	0.317	
Behavior	50	0.14	1.13	6, 92	0.35	
Executive Function	68	0.22	1.25	12, 122	0.257	
Motor	79	0.31	3.43	8, 148	0.001	**
Sensory	78	0.33	1.73	16, 138	0.047	*

Multivariate analysis of variance (MANOVA) was performed to compare behavioral measures across SKS, PTEN-ASD, and macrocephaly-associated ASD groups. N indicates number of subjects with complete data for all measures within each domain. Pillai: Pillai's trace statistic; F: F-statistic; df: degrees of freedom. Domain measures as defined in Table 1. *p<0.05, **p<0.01, ***p<0.001.

Table S7. Kruskal-Wallis Test Results for Neuropsychological Measures Across MTOR/PTEN Domains

Phenotype	Chi-squared	df	p-value	Significance
DCDQ Fine Motor	20.461118	3	0.000136	***
DCDQ Total Score	12.483682	3	0.005897	**
SRS-2 Total T-Score	11.328336	3	0.010077	*
VABS-2 Daily Living Skills	11.251485	3	0.010441	*
ABC Irritability	9.911848	3	0.019331	*
RBSR Total Score	9.671468	3	0.021575	*
CBCL Externalizing	9.506216	3	0.023265	*
ABC Stereotypy	9.252974	3	0.02611	*
BRIEF-P Emotional Control	8.945154	3	0.030029	*
VABS-2 Communication	8.881718	3	0.030906	*
CBCL Total	8.69116	3	0.033692	*
VABS-2 Adaptive Behavior Composite	8.509843	3	0.03657	*
BRIEF-P Working Memory	8.444515	3	0.037665	*
DCDQ Control During Movement	8.354609	3	0.039224	*
DCDQ General Coordination	8.286289	3	0.040451	*
BRIEF-P Organization of Materials	7.87418	3	0.048685	*
ABC Total Score	7.834389	3	0.049561	*
CBCL Internalizing	7.160743	3	0.066947	ns
BRIEF-P Global Executive Composite	6.329578	3	0.096631	ns
BRIEF-P Behavioral Regulation Index	6.256085	3	0.099795	ns
VABS-2 Motor Skills	6.225118	3	0.101157	ns
BRIEF-P Initiate	6.223361	3	0.101235	ns
ABC Lethargy	6.038411	3	0.109756	ns
ABC Hyperactivity	4.713158	3	0.194047	ns
VABS-2 Socialization	4.623689	3	0.201519	ns
BRIEF-P Metacognition Index	4.546817	3	0.208152	ns
ABC Inappropriate Speech	4.482657	3	0.213842	ns
BRIEF-P Shift	4.481134	3	0.213979	ns
BRIEF-P Plan/Organize	3.618187	3	0.305754	ns
BRIEF-P Inhibition	3.221903	3	0.358661	ns
BVMI Standard Score	1.028643	2	0.597906	ns
BRIEF-P Monitor	1.843478	3	0.60552	ns

The table presents the results of Kruskal-Wallis tests performed to compare different protein domains across various neuropsychological measures. Significance levels are indicated as follows: * $p<0.05$, ** $p<0.01$, *** $p<0.001$, ns not significant. **Abbreviations**: VABS, Vineland Adaptive Behavior Scales; SRS, Social Responsiveness Scale; CBCL, Child Behavior Checklist; BRIEF-P, Behavior Rating Inventory of Executive Function - Preschool Version; ABC, Aberrant Behavior Checklist; DCDQ, Developmental Coordination Disorder Questionnaire; BVMI, Beery-Buktenica Developmental Test; df, degrees of freedom.

Table S8. Pairwise Comparison Results for Neuropsychological Measures Across MTOR/PTEN Domains

Phenotype	PTEN p-value	PTEN N	MTOR p-value	MTOR N	PTEN Sig	MTOR Sig
SRS-2 Total T-Score	0.007596	39	0.828139	15	**	ns
BRIEF-P Organization of Materials	0.009822	30	1	5	**	ns
RBSR Total Score	0.01089	41	0.47009	15	*	ns
BRIEF-P Working Memory	0.017942	31	1	5	*	ns
CBCL Internalizing	0.020323	21	1	15	*	ns
CBCL Total	0.022851	21	0.217806	15	*	ns
BRIEF-P Initiate	0.03487	30	0.723674	5	*	ns
BRIEF-P Global Executive Composite	0.041655	30	0.723674	5	*	ns
BRIEF-P Emotional Control	0.044027	31	0.723674	5	*	ns
BRIEF-P Metacognition Index	0.049685	30	1	5	*	ns
ABC Inappropriate Speech	0.066253	42	0.764503	15	ns	ns
CBCL Externalizing	0.081317	21	0.168398	15	ns	ns
BRIEF-P Plan/Organize	0.121951	30	0.288844	5	ns	ns
ABC Lethargy	0.122451	42	0.217391	15	ns	ns
BRIEF-P Behavioral Regulation Index	0.12786	30	0.716801	5	ns	ns
ABC Stereotypy	0.179936	42	0.611838	15	ns	ns
BRIEF-P Shift	0.217766	31	0.2765	5	ns	ns
VABS-2 Daily Living Skills	0.227381	39	1	12	ns	ns
VABS-2 Adaptive Behavior Composite	0.271528	38	0.360765	10	ns	ns
ABC Total Score	0.277263	41	0.942365	15	ns	ns
BRIEF-P Inhibition	0.553848	31	0.288844	5	ns	ns
VABS-2 Communication	0.34767	39	0.931608	13	ns	ns
BRIEF-P Monitor	0.358517	29	1	5	ns	ns
VABS-2 Socialization	0.37005	38	0.757315	11	ns	ns
ABC Irritability	0.40207	42	0.942209	15	ns	ns
BVMI Standard Score	0.467631	40	NA	2	ns	ns
DCDQ Control During Movement	0.657687	39	0.581739	15	ns	ns
DCDQ General Coordination	0.592097	39	0.706165	15	ns	ns
DCDQ Fine Motor	0.700615	39	0.738883	15	ns	ns
ABC Hyperactivity	0.758291	42	0.884722	15	ns	ns
VABS-2 Motor Skills	0.819499	18	1	9	ns	ns
DCDQ Total Score	0.927195	39	0.88325	15	ns	ns

Pairwise domain comparisons of neuropsychological scores. The table presents pairwise Wilcoxon test results comparing PTEN domains (Phosphatase vs C2) and MTOR domains (FAT vs PI3K) for each neuropsychological measure. Sample sizes (n) and significance levels (* $p<0.05$, ** $p<0.01$, ns not significant) are shown for each comparison. **Abbreviations**: VABS, Vineland Adaptive Behavior Scales; SRS, Social Responsiveness Scale; CBCL, Child Behavior Checklist; BRIEF-P, Behavior Rating Inventory of Executive Function - Preschool Version; ABC, Aberrant Behavior Checklist; DCDQ, Developmental Coordination Disorder Questionnaire; BVMI, Beery-Buktenica Developmental Test.

Figure S1. Domain-Specific Effects on Additional Neuropsychological Phenotypes

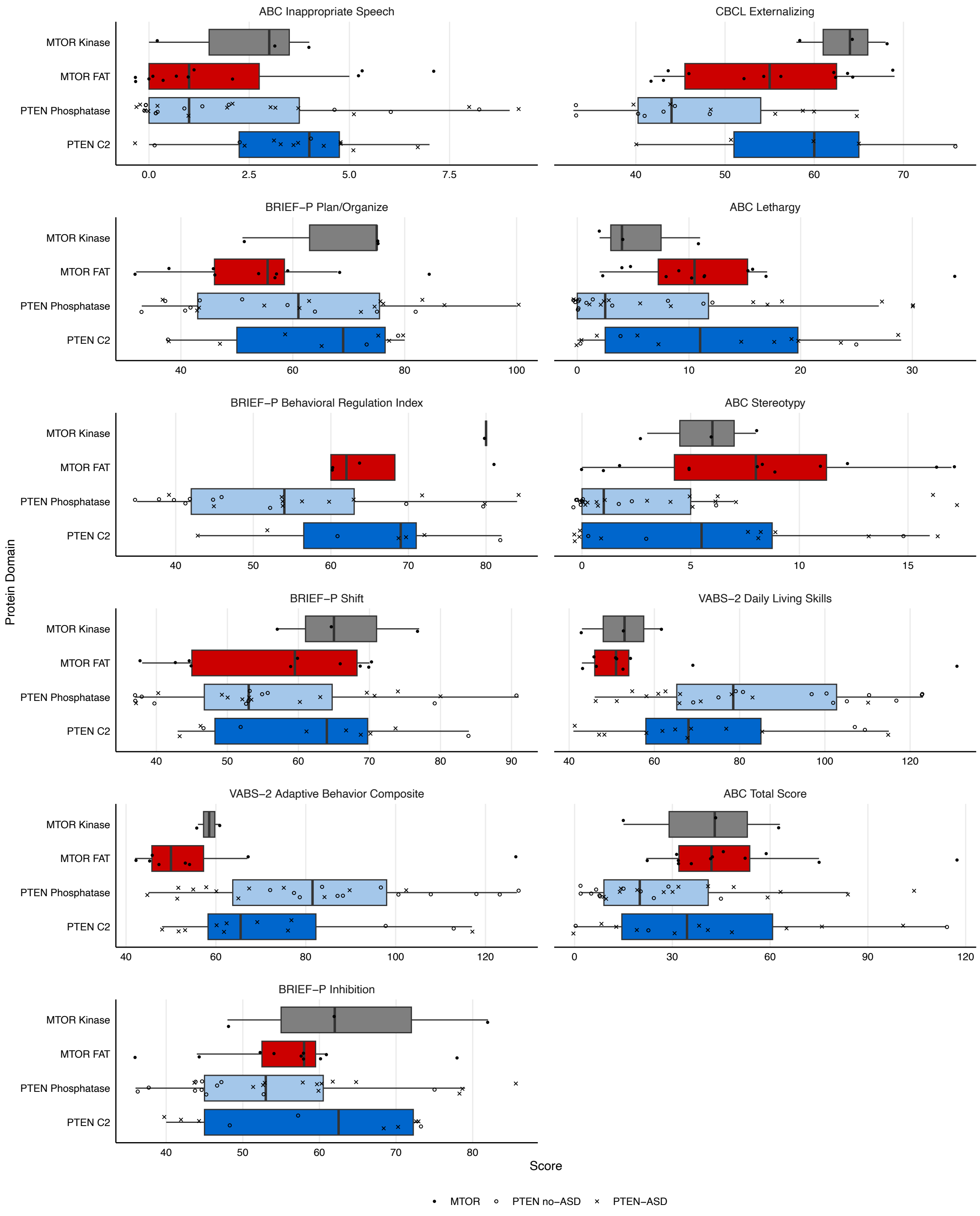

Figure S1. Continued

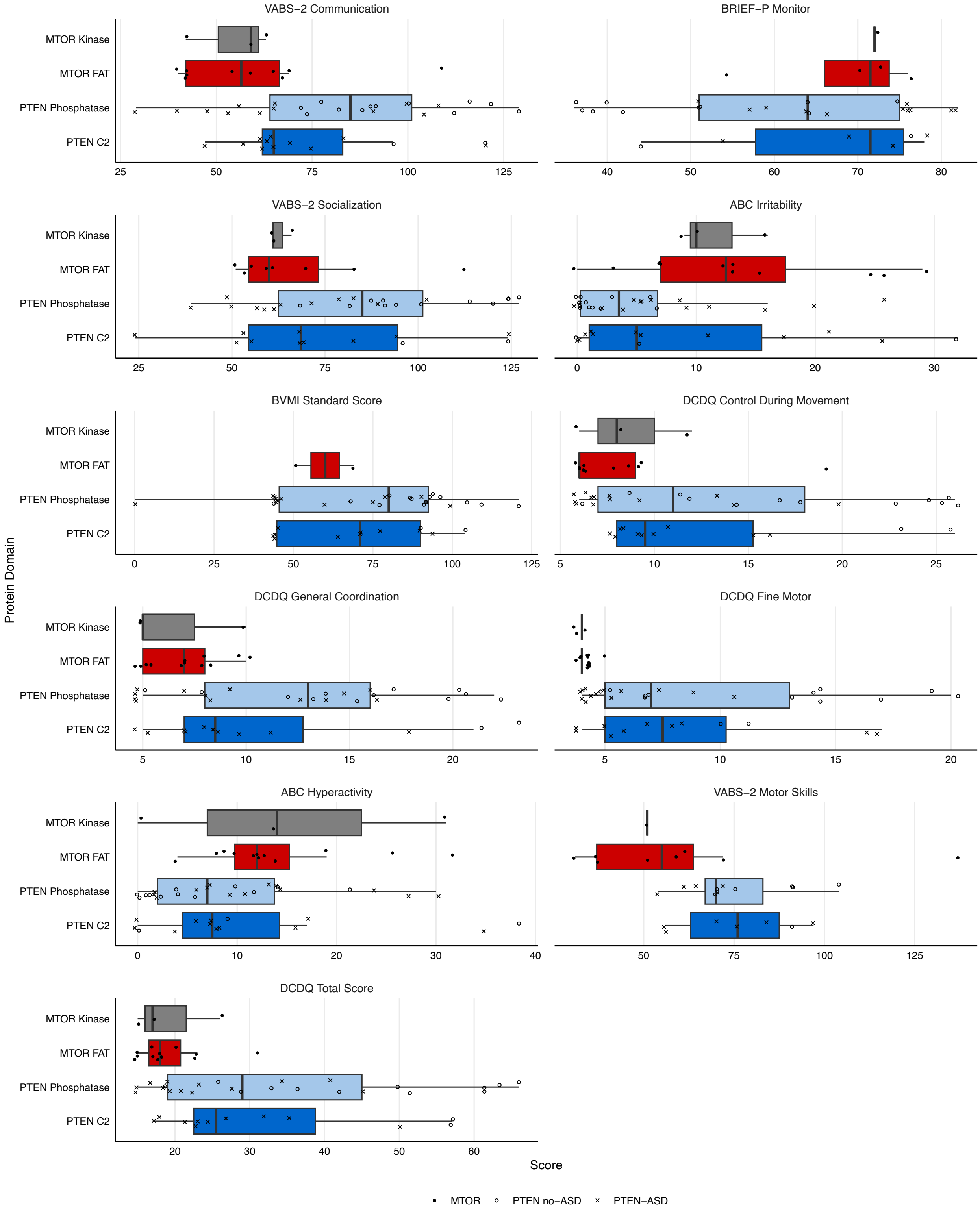

Domain-specific differences in additional neuropsychological phenotypes. Box plots showing the distribution of scores for the remaining twenty-two neuropsychological measures across PTEN

and MTOR protein domains. Box plots represent median and interquartile range, with individual data points overlaid. For PTEN variants, filled circles indicate individuals with ASD diagnosis while X's indicate those without ASD. Diamond shapes represent individuals with MTOR variants. Colors indicate protein domains: PTEN Phosphatase (light blue), PTEN C2 (dark blue), MTOR FAT (light red), and MTOR PI3K (dark red). Abbreviations: ABC, Aberrant Behavior Checklist; BVMI, Beery-Buktenica Developmental Test; DCDQ, Developmental Coordination Disorder Questionnaire; VABS, Vineland Adaptive Behavior Scales; BRIEF-P, Behavior Rating Inventory of Executive Function - Preschool Version.
